# Nanomedicine Approaches for Corneal Diseases

**DOI:** 10.3390/jfb6020277

**Published:** 2015-04-30

**Authors:** Shyam S. Chaurasia, Rayne R. Lim, Rajamani Lakshminarayanan, Rajiv R. Mohan

**Affiliations:** 1 Harry S. Truman Memorial Veteran Hospital, Columbia, MO 65201, USA; 2 Department of Ophthalmology Veterinary Medicine & Surgery, Columbia, MO 65211, USA; E-Mails: chaurasias@missouri.edu (S.S.C.); rayne.ruiyi.lim@gmail.com (R.R.L.); 3 Mason Eye Institute, University of Missouri-Columbia, Columbia, MO 65211, USA; 4 Singapore Eye Research Institute, 20 College Road, 169856, Singapore

**Keywords:** corneal diseases, infection, scarring, fibrosis, neovascularization, nanoparticles, nanomedicine, nanomaterials, nanodelivery

## Abstract

Corneal diseases are the third leading cause of blindness globally. Topical nonsteroidal anti-inflammatory drugs (NSAIDs), steroids, antibiotics and tissue transplantation are currently used to treat corneal pathological conditions. However, barrier properties of the ocular surface necessitate high concentration of the drugs applied in the eye repeatedly. This often results in poor efficacy and several side-effects. Nanoparticle-based molecular medicine seeks to overcome these limitations by enhancing the permeability and pharmacological properties of the drugs. The promise of nanomedicine approaches for treating corneal defects and restoring vision without side effects in preclinical animal studies has been demonstrated. Numerous polymeric, metallic and hybrid nanoparticles capable of transporting genes into desired corneal cells to intercept pathologic pathways and processes leading to blindness have been identified. This review provides an overview of corneal diseases, nanovector properties and their applications in drug-delivery and corneal disease management.

## 1. Introduction

The cornea is a highly specialized sensory, avascular, transparent connective tissue that serves as a mechanical barrier and provides two-third refractive properties of the eye [1,2,3]. The cornea comprises mainly three layers of cells: the outermost epithelial cells that act as a barrier to the threats from the outside environment, the middle layer corneal stroma that constitutes 90% of the cornea and contains highly organized collagen fibrils, proteoglycans and keratocytes, and the single layer of endothelial cells that constitutes the innermost layer of the cornea [4,5,6]. 

Corneal diseases are either congenital or acquired through injury, trauma, infection and/or surgery to the eye, resulting in the loss of transparency and visual dysfunction [7,8,9,10]. In physiological conditions, corneal insult triggers a complex wound healing response to retain corneal integrity and maintain normal corneal structure and transparency. However, in pathological conditions, cornea can elicit excessive wound healing and long-term deleterious effects because of hyperactivity of cytokines, growth factors and chemokines. This results in corneal epithelial–stromal interactions and extracellular matrix (ECM) remodeling, myofibroblast generation, decrease in cellular corneal crystallins and loss of stromal structural components [11,12,13,14]. 

Current therapeutic strategies include topical administration of antibiotics, steroids, nonsteroidal anti-inflammatory drugs (NSAIDs), phototherapeutic surgery (PTK) and transplantation of full thickness cornea (PK). Topical eyedrops are the most commonly used method of medication for the prevention and intervention of corneal defects. Nevertheless, these drugs are often ineffective due to poor patient compliance, low penetration through the epithelial barrier and unwanted side-effects [15,16,17,18]. Over the last two decades, newer treatment methods have been adopted for corneal diseases including limbal stem cell transplantation, specific corneal cells/tissue transplantation (DSAEK, DMEK), drug release via contact lens and gene therapy techniques. Although corneal transplantation is often used in the management of the corneal diseases, insufficient supply of donor corneal tissues and considerable rejection rates have warranted the development of new approaches for corneal disease treatment and management. 

Nanomedicine is a medical application of nanotechnology, nanodevices and nanomaterials for tissue repair and drug delivery for the treatment of human diseases. It utilizes materials with a dimension of 1–100 nm to function at the cellular and molecular levels. In the last decade, nanomedicine has evolved dramatically in various commercial and scientific fields, from consumer goods to cosmetics, chemistry and agriculture sciences to pharmacology and medical sciences [19,20,21]. Nanomedicine has provided newer diagnostic tools and promising therapies for a variety of scientific fields including ophthalmology [22,23,24]. In the field of cornea research, nanomedicine has been particularly focused on imaging, preventing and/or reducing corneal opacities and neovascularization [25,26,27,28]. This review highlights the recent advances in the nanomedicine approaches for the treatment of vision-impairing corneal diseases.

## 2. Pathology of Corneal Diseases

Corneal diseases refer to a variety of disorders caused by inflammatory, infectious, degenerative and traumatic conditions [9,10]. Proper healing of the cornea following insult to the eye is vital for maintaining a transparent and nonvascular cornea required for normal vision [11,12,13,14]. Thus, molecular mechanisms mediating corneal wound healing are of critical importance in corneal disease pathology, not only to ensure the integrity of the eye but also to maintain the best possible vision.

### 2.1. Corneal Infection 

The cornea is often under attack by micro-organisms [29]. Antimicrobial proteins/peptides endogenously present in the tear film provide the first line of defense against the invading microorganisms and also regulate wound healing [30]. Lysozyme, lactoferrin, phospholipase A2, defensins, histatins, and cathelidins are the key components of the host defense system [31,32,33]. A decrease in concentrations of the antimicrobial proteins or alterations in the protective tear coating and surface epithelia cells could potentially lead to infections such as microbial keratitis (MK) [34]. The most common etiological agents are bacteria, fungus, protozoa and parasites. Common risk factors for MK include ocular trauma, contact lens wear, topical anesthetics/corticosteroids, neurotrophic disease, lid or lash malposition, tear insufficiency, stem cell deficiency and systemic abnormalities [35,36,37]. 

Topical application of antibiotic eye drops is the common route of administration for MK. Topical delivery of antibiotics is effective, however, is often challenging due to patient compliance and toxicity. Moreover, the topical application of drugs in MK is compromised due to poor drug penetration (generally <5%) and bioavailability in ocular tissues. Furthermore, other factors such as blinking reflex and tear turnover pose additional challenges [38]. It is also observed that a significant amount of topical drugs drain into the nasal cavity or accumulate in the nasolacrimal system, which may cause systemic side effects [39].

### 2.2. Corneal Scarring/Fibrosis 

Scarring or fibrosis leads to loss of transparency in the cornea, which is critical for sharp vision. Corneal epithelium is the first line of defense against injury and has the intrinsic capacity to renew every three to four days to maintain its barrier function [4,40]. In the case of minor injury or scratches, healthy epithelial cells migrate to patch the injured area and vision is unaffected. If the scratch penetrates into the stroma, the healing process lengthens. This could at times result in pain, blurred vision, tearing, redness and sensitivity to light. If left untreated or not treated well, deeper scratches cause corneal scarring resulting in a haze [41,42,43]. 

**Figure 1:**
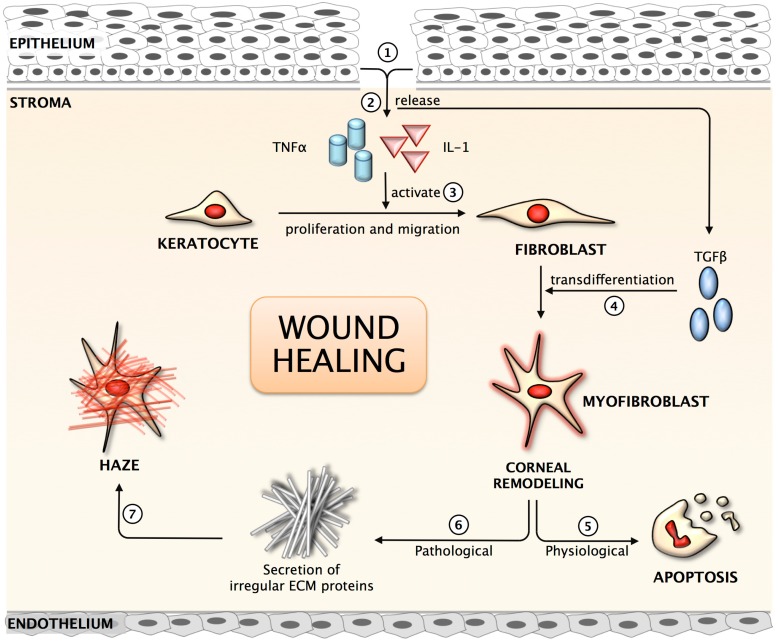
Schematic representation of the corneal wound healing mechanism. (1) Corneal injury results in the loss of basement membrane; (2) Release of pro-inflammatory cytokines into the anterior stroma; (3) Activation of quiescent keratocytes to fibroblast; (4) Growth factor released from the epithelium & TGFβ result in trans-differentiation of fibroblast to myofibroblast, the repair phenotype; (5) Under normal physiological condition, myofibroblasts undergo apoptosis following repair to the cornea; (6) In pathological conditions, myofibroblasts secrete irregular matrix; (7) Clinical observation of corneal haze in the anterior stroma.

**Figure 2:**
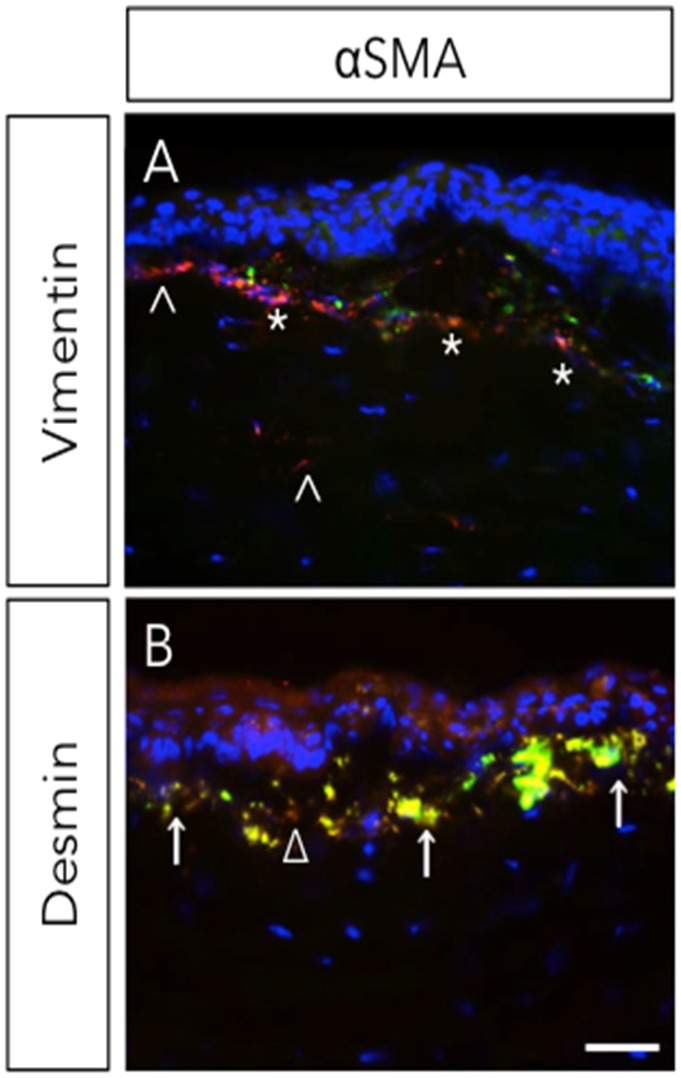
Expression of vimentin and desmin during corneal wound healing. Rabbit underwent −9D photorefractive keratectomy (PRK). Corneas were stained with αSMA (myofibroblast marker) with vimentin (**A**) or desmin (**B**) four-week post-surgery. ^, vimentin+; *, vimentin+ & αSMA+; Δ, desmin+; ↑, desmin+ & αSMA+. Scale bar = 25 μm. Reprinted from [14]. Copyright Elsevier 2009.

**Figure 3:**
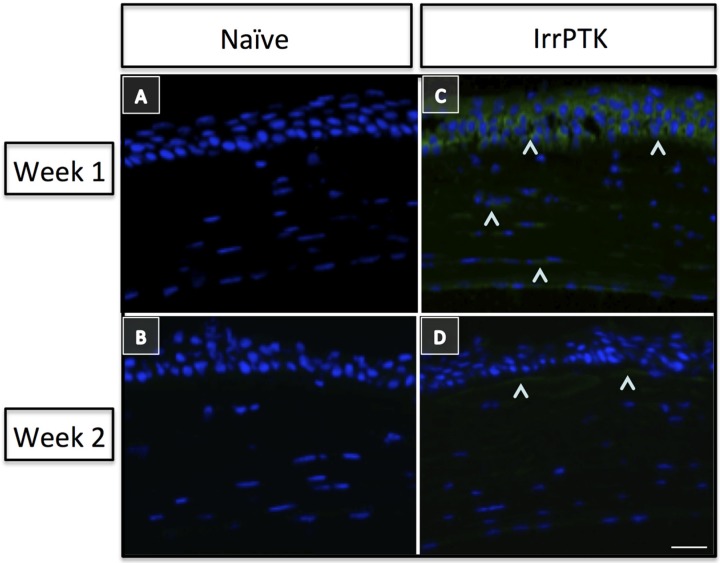
Hevin plays a critical role in corneal wound healing. Hevin is not expressed in C57BL/6J naïve mouse cornea (**A**,**B**). Hevin (^) is upregulated in irregular phototherapeutic keratectomy (IrrPTK) mice one week post-surgery (**C**) and decrease in expression of Hevin two weeks post-surgery (**D**). Scale bar = 25 μm. Reprinted from [47]. Copyright PLOS 2013.

Stromal wound healing in the cornea is a complex process (Figure 1), controlled by the interactions and signaling between epithelial and stromal cells. Keratocytes, present beneath the epithelium in the corneal stroma, exhibit relatively low levels of activity and are considered quiescent in the adult cornea [44,45,46]. Insult to corneal stroma triggers inflammatory response, cell proliferation, and secretion of several growth factors, chemokines, extracellular and matricellular proteins. The activation of inflammatory cells close to the site of wound, which are cleared by apoptosis in the initial phase of wound healing, limits the inflammatory response and loss of intracellular components, thus demonstrating effective wound healing. A previous study suggests that vimentin+ and desmin+ stromal cells play an essential role in the early and intermediate stages of the formation of myofibroblasts (Figure 2) during corneal wound healing [14]. These myofibroblasts produce high levels of collagen, hyaluronan and biglycan to form a disorganized and opaque cornea. Several matrix metalloproteases (MMPs) and tissue inhibitors of matrix metalloproteases (TIMPs) are released during wound healing and they contribute in matrix remodeling by removing irregular matrix and reinstating newer ECM. Recently, it has been reported that Hevin, a matricellular protein, plays a role in the modulation of corneal wound healing in a mouse model [47]. It is transiently expressed in the early stages of corneal wound healing and its functional loss predisposes injured cornea to chronic inflammation and fibrosis (Figure 3). Thus, proper disposal of the transient matrix and its replacement by organized and mature ECM (with matricellular proteins) forms an integral part of the corneal transparency. Any disorganization or the non-removal of the degenerate matrix can lead to aberrant wound healing in the corneal stroma and, hence, impaired vision. 

The present strategy to treat corneal scarring/fibrosis depends on the severity of the symptoms. Current treatment involves topical antibiotics, steroids, anti-scarring drugs and surgical procedures. Unfortunately, the commonly used topical drugs such as corticosteroids and cyclosporins are variably effective in patients and often carry severe side effects like cataract and glaucoma.

### 2.3. Corneal Neovascularization 

Corneal neovascularization is a sight-threatening condition commonly caused by inflammation, infection, chemical injury, autoimmune conditions, post-corneal transplantation and traumatic conditions. The most important aspect of corneal pathophysiology is the maintenance of an avascular stroma throughout the lifespan of the normal cornea. Several molecules and growth factors contribute to the corneal avascularity to prevent corneal neovascularization; one of the most important growth factors being the pigment epithelium-derived factor (PEDF) [48]. PEDF has been shown to inhibit PEDF-derived peptide, decrease VEGF expression and inhibit corneal neovascularization [49]. Several studies have suggested that soluble(s)Flt-1, an isomeric soluble form of VEGFR-1, plays an important role in maintaining the avascularity of the corneal tissue [50]. sFlt-1 is known to sequestrate VEGF ligands, thus neutralizing the angiogenic effects of VEGF in the cornea. Similarly, thrombospondins (TSPs), a family of endogenous angiogenic inhibitors, are present in the cornea and inhibit neovascularization in the stroma [51]. Several other molecules including angiopoietin-like molecule, cornea-derived transcript-6 (CDT6) [52] and the inhibitory PAS-domain transcription factor (IPAS) have also been reported to inhibit corneal neovascularization [53]. 

The cornea is considered as an immune privileged tissue under a tight control of local pro- and anti-angiogenic factors [54,55]. Corneal neovascularization is induced in the inflammatory or hypoxia conditions by a cross-talk between the epithelial and stromal cells that results in the up-regulation of angiogenic factors [56,57]. The pathogenesis of neovascularization involves multiple growth factors, cytokines, chemokines and immune cells influenced by matrix metalloproteinases and other proteolytic enzymes. In addition, macrophages are recruited by the invading endothelial cells to produce pro-angiogenic factors such as VEGF, MIF and bFGF [58,59]. While MIF increases the angiogenic response, bFGF stimulates the proliferation and migration of endothelial cells [59]. These events lead to lipid deposition, stromal edema, tissue scarring and stromal hemorrhage resulting in the significant reduction in visual acuity. 

Current treatments for corneal neovascularization include topical corticosteroid and NSAIDs photodynamic therapy, laser photocoagulation and tissue transplantation [60,61,62]. Although therapeutic advances in the treatment of corneal neovascularization have been made with corticosteroids, they do not always inhibit neovascularization. In addition, there are always increased cost-related issues tagged to tissue transplantation and often comes with serious side effects including elevated intraocular pressure, posterior subcapsular cataracts and glaucoma. Therefore, alternative therapeutic strategies that could target molecular mediators of angiogenesis and improvised drug delivery methods are needed.

## 3. Nanomedicine for Corneal Diseases 

**Figure 4:**
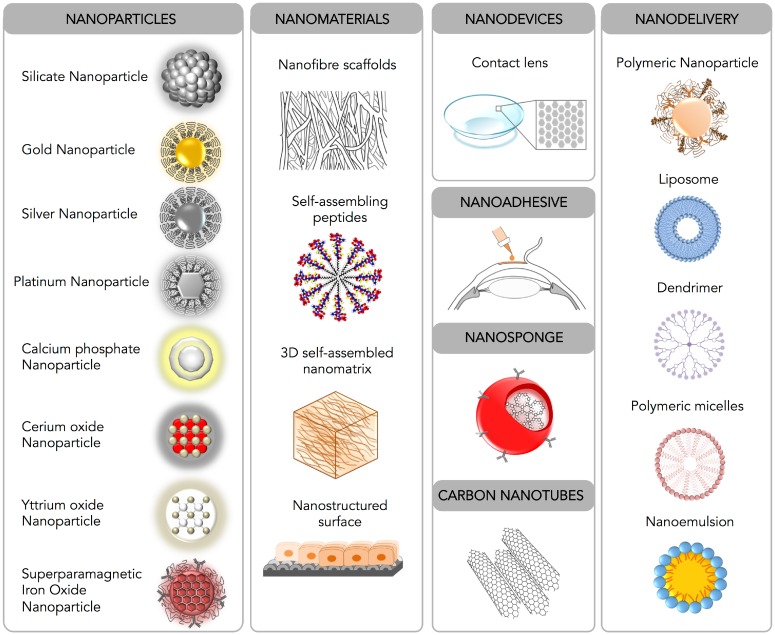
Schematic diagram depicting nanomedicine techniques available for corneal diseases.

Nanomedicine is an emerging field of medical science which utilizes nanotechnology to study the functioning of the living cells at the molecular level and nanomaterials to develop newer drug delivery modalities for the treatment of human diseases. Nanotechnology has been used in almost every field of medical science including imaging, diagnosis, biosensors and drug delivery. In the following section, current nanomedicine tools that have been tested for the treatment of corneal diseases are described (Figure 4).

### 3.1. Nanoparticles

Nanoparticles ranging from 1 to 100 nm are widely useful for the nanomedicine due to their (i) small size, (ii) ability to enter the intracellular compartment of the cell, (iii) high surface-area-to-volume ratio, (iv) capacity to engage and deliver large payload and (v) minimal toxic damage to cell membranes and the cellular environment. 

Nanoparticles are highly suited for treatment of eye diseases as they can pass through the physical barriers of the various tissues including cornea, conjunctiva, sclera and in some cases blood–retinal barriers. Most importantly, multiple ligands such as DNA, antibodies, peptides, molecular sensors, therapeutic molecules and probes can be loaded onto the nanoparticles and transported into desired cells of the eye. The utility of nanoparticles in the treatment of corneal diseases has been recently demonstrated [25,26,27,28,63]. 

Nanoparticles are broadly classified into metallic, polymeric and hybrid nanoparticles. Metallic nanoparticles include gold (Au-NPs), sliver (Ag-NPs) and platinum (Pt-NPs). Gold and silver nanoparticles are commonly used as biocarriers because they are inert, cost-effective, easy to make and non-toxic in the cellular systems [64]. They can be cargoed and successfully expressed into the mammalian cells [65,66]. Recently, platinum nanoparticles have shown anti-ageing properties [67] but have not yet been tested on eye. The polymeric nanoparticles are usually prepared from polyethyleneimine (PEI), albumin, chitosan and polyethylene glycol. They have been reported to deliver transgene into human corneal epithelial cells and endothelial cells *in vitro* [68,69] and efficiently deliver genes in rodent corneas *in vivo* without significant side effects [68,69,70,71]. However, some polymeric formulations such as Poly (d,l-lactide-*co*-glycolic acid) (PLGA) nanoparticles were not found to be efficacious in the cornea [72].

**Figure 5:**
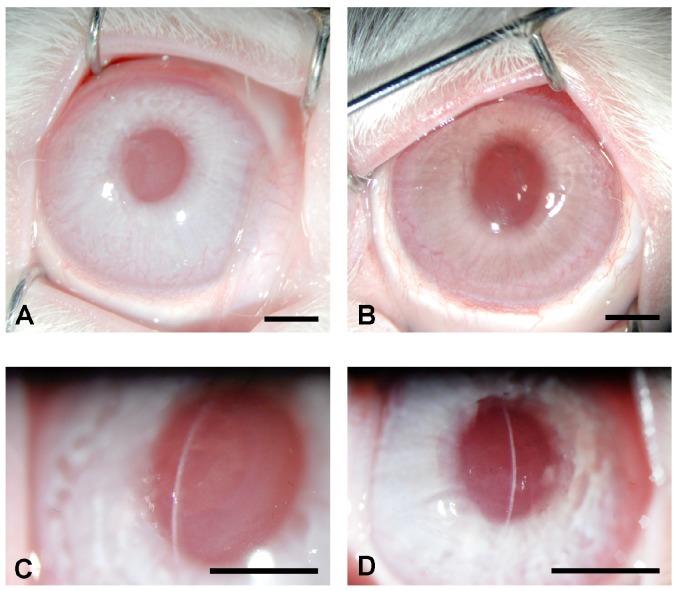
Treatment of corneal haze with nanoparticles. Corneal haze was developed in rabbit corneas using −9.0 diopter photorefractive keratectomy (PRK) with excimer laser. Representative stereomicroscopy (**A**,**B**) and slit-lamp (**C**,**D**) images of laser-ablated rabbit corneas that received a single 5 min topical application of PEI2-AuNPs nanoparticle transfection solution without BMP7 (**A**,**C**) or with BMP7 expressing gene (**B**,**D**) obtained four weeks after PRK.

Hybrid nanoparticles are the most widely used metallic nanoparticles conjugated with polymeric compounds studied in ophthalmology. Recently, the efficiency of 2kDa PEI conjugated to gold nanoparticles (PEI2-Au-NPs) for delivering genes in the human cornea *in vitro* and rabbit cornea *in vivo* has been reported (Figure 5). This was the first report of hybrid nanoparticles delivering foreign genes into the rabbit cornea *in vivo* with a low toxicity, rapid uptake and slow clearance, suggesting that PEI2-AuNPs may provide a safe and effective platform for delivering therapeutic genes into desired corneal cells [25]. These nanoparticles can bind large therapeutic genes, which make them an excellent candidate for corneal nanomedicine development [25]. 

Non-metallic nanoparticles such as calcium phosphate nanoparticles (CaP-NPs) functionalized with pcDNA3-EGFP (CaP/DNA/CaP/PEI0.5) have been shown to be an effective tool for transfection in cells because of their high biocompatibility and easy biodegradability. Once transfected in human and murine corneal endothelial cells, they dissociate into calcium and phosphate ions for clearance. The CaP-NPs successfully transfected corneal endothelial cells with moderate toxicity and increase in intracellular calcium [73].

### 3.2. Nanofiber Scaffolds 

For ocular nanomedicine, nanofiber scaffolds such as self-assembling peptides that provide framework and optimal conditions for the cells and tissue regeneration present a huge promise. These scaffold-like tissue-bridging nanostructures were successfully used in the treatment of blindness in animal models [74,75]. In these studies, new or improved materials were prepared based on the molecular self-assembly at the same scale observed in intracellular molecules and structures. One such example is the preparation of nanostructured surface termed as cell-sheet engineering approach to culture corneal endothelial cells under optimal conditions [76]. This can be further improvised by adding therapeutics to the three-dimensional nanostructures before implanting to the eye. Recently, Ma *et al.* (2013) in a similar study used PLGA as the scaffold for the preparation of rabbit adipose derived stem cells for corneal transplantation [77]. The PLGA scaffold not only provided a bed for the differentiation of these cells to functional corneal keratocytes, but also repaired the corneal stromal defects. Thus, nanostructured scaffolds may provide an ideal surface for cell adhesion and migration which can likely decrease the chances of rejection. Octaamine dendrimers coupled with polypropyleneimine have been cross-linked by collagen to produce a novel tissue-engineering scaffold of high mechanical strength for corneal cell growth and adhesion with no cell toxicity [78]. Recently, some of these nanomaterials have been tried in clinical trials and approved by the FDA for use in humans [79,80,81]; many of them are either in proof-of-concept studies for cell culture or small animal models for medical applications [82,83,84].

### 3.3. Nanodevices

Nanodevices have great potential in treating blinding disorders. In recent years, there has been a surge in the development of nanodevices for sustained drug delivery and improved ocular surgeries. The most common nanodevices are soft lens containing drugs and are designed to release a therapeutic amount of drug over a long period of time [85,86]. The nanospheres made by pullulan and polycaprolactone that contain ciprofloxacin coated on to contact lenses are the best example of nanodevices [87]. These contact lenses successfully prevent Staphylococcus aureus and Pseudomonas aeruginosa infection due to sustained release of ciprofloxacin for extended durations. Recently, molecular imprinting of the contact lenses has emerged as another promising nanodevice for sustained drug delivery. These lenses are believed to be superior to earlier drug-soaked contact lens nanodevices because of the presence of high affinity binding sites to the specific drugs that provide more reproducible and controlled release of the drugs [88,89,90]. However, these nanodevices have not yet made their way to the clinics due to high costs, discomfort to the patients and issues associated with drug clearance*.*

### 3.4. Nanoadhesives, Nanosponges and Carbon Nanotubes 

The biomimetic materials used in tissue engineering were utilized to generate biocompatible nanoadhesive to heal, seal and repair ocular tissues. They allow cells to adhere, grow and proliferate during wound healing [91]. The clinical utility of nanoadhesives is yet to be tested. Nanosponges are synthesized by crosslinking of β-cyclodextrins with diphenyl carbonate to construct a colloidal system with a high efficiency hyper-branched polymer [92]. This system provides an excellent solubility and corneal penetration for drugs such as dexamethasone that generally exhibit poor solubility and permeability through the ocular surface [93]. The experiments on bovine cornea showed that nanosponges are highly permeable and safe to corneal epithelial cells. Carbon nanotubes (CNTs), miniaturized cylindrical carbon structure, are widely used in the fields of electronics, energy, environment, material science and healthcare. Ever since its inception, single-/multiple-walled CNTs have gained considerable attention due to their exceptional mechanical, optical, electrical and thermal properties [94]. These attractive properties have been successfully employed to fabricate composites, sensors, energy storage devices and microelectronics [95]. The high surface areas and reactivity of the surfaces of CNTs provide both non-covalent and covalent functionalization of the drugs and fluorescence probes, thus expanding its potential as a drug carrier as well as for diagnostic purposes [96]. The potential of CNTs in corneal disease management is yet to be investigated.

### 3.5. Nanodelivery 

One of the most important advances in the field of ocular nanomedicine was attained by the design and development of nanoparticle-based drug delivery systems [97]. It is anticipated that such newer delivery system will be free from many drawbacks of the conventional ophthalmic drops including less than 5% drug absorption and repeated application [98]. Nanotechnology-based drug delivery systems in the eye showed increased drug efficacy, reduced dosage and application, high bioavailability, sustained release and less systemic effects [99,100,101,102]. The pilocarpine-loaded nanospheres using a poly (methylmethacrylate-acrylic acid) copolymer delivery system has shown benefit in reducing high intra-ocular pressure [103]. Nanodelivery methods can be broadly classified based on drug packaging as polymeric nanoparticles, liposomes, dendrimers, or nanoemulsions. 

Polymeric nanoparticles (PNs) are colloidal carriers with diameters ranging from 10 to 1000 nm. PNs have been widely studied as topical ocular drug delivery systems because of their enhanced adherence to the ocular surface and their controlled release of drugs [104,105]. PN systems allow a greater amount of design flexibility in terms of the size, surface charge and composition to improve drug penetration, retention time and sustain drug delivery. In addition, they can be formulated and administrated as eye drops which make them ideal candidates for the treatment of corneal diseases. Some of the examples are polymers such as polylactide (PLA), PLGA, poly 3-caprolactone (PCL) and PEI-conjugated nanoparticles have been successfully exploited using the polymeric nanocolloid systems [106,107,108].

Recently, Tandon and co-workers showed using an *in vivo* rabbit model of laser ablation-induced corneal fibrosis that polyethyleneimine-conjugated gold nanoparticles (PEI2-Au-NPs)-mediated BMP7 gene therapy inhibits corneal haze through a counter balancing TGFβ1-mediated profibrotic Smad pathway [28]. Another study by Chowdhary *et al.* using pirfenidone loaded PLGA nanoparticles reported decreased collagen synthesis, reduced myofibroblast formation and improved corneal wound healing treated in corneal alkali burn model [27]. Qazi *et al.* designed a small hairpin RNA (shRNA) expressing plasmid encapsulated in PLGA nanoparticles to inhibit angiogenesis in a mouse model of corneal neovascularization [109].

Liposomes are composed of one or more phospholipid bilayer membranes encapsulating a volume of aqueous medium and are classified based on the size and the number of bilayers [110]. In contrast to other delivery systems that deliver the drugs at the site of infections/injury, liposomes deliver the active drugs to the target cells in addition to the wounded sites. Depending on the nature of the drugs and intended applications, the lipid composition, liposome size, membrane fluidity and surface charges can be modified to increase the therapeutic efficacy. The first investigation which describes the use of liposomes for the topical delivery of ocular drugs was described by Smolin *et al.* where they used a liposomal formulation of idoxuridine to improve the efficacy of this drug for the treatment of herpes keratitis [111]. Various liposomal formulations of antimicrobials have also been used for the delivery of drugs into the cornea. The use of liposomes is attributed to enhance the absorption of the formulations as well as optimal release of the encapsulated drug. Since the corneal surface is negatively charged, the presence of positively charged lipids in the liposomes enhanced the retention of the drugs in the cornea by forming a coated surface for topical applications [112]. Although liposomes have shown increased retention, corneal penetration and sustained drug delivery, its use has been limited in the corneal diseases due to instability, degradability, aggregation of the liposomes and limited drug-loading capacity [113,114,115]. Table 1 summarizes the efficacy of liposomal formulations in transcorneal permeation as well as *in vivo* efficacy compared to free drug formulations.

Dendrimers are three-dimensional and hyper branched nanostructures that have widely used in several applications including gene therapy, bioimaging and drug delivery [116,117,118]*.* They are typically 1–10 nm in size and can be precisely complexed, conjugated or encapsulated to control the dendrimer shape and surface functionality for sustained drug delivery [119,120]. The first known dendrimer, Poly(amidoamine) (PAMAM) was synthesized by Tomalia *et al.* in the 1980s [121]. Since then, PAMAM have been widely used in the drug delivery system due to its ease in synthesizing, stability and low cytotoxicity to cells [122]. Dendrimers have also been used in ocular drug delivery where PAMAM was found to increase drug residence time, corneal penetration and bioavailability compared to the free drug in the solution [123]. Dendrimers packaged with antimicrobial agents have been found to be effective against gram-negative and gram-positive pathogens often associated with lens-related bacterial keratitis [124]. However, they have been reported to cause blurred vision in animal models.

Polymeric micelles (PMs) are self-assembled nanoparticles. They are characterized by a unique core-shell structure containing hydrophobic depot for the therapeutic drug whereas the hydrophilic shell interacts with the core solvents to provide long-term stability to the drug [125,126]. PMs are usually biodegradable and biocompatible, ideally suited for ocular drug delivery. Several studies have shown their ability to cross corneal surface barriers and improve permeability of the ocular drugs [127,128]. Thus, PMs have several advantages over the conventional eye drops in terms of stability and sustained release of drug for the treatment of corneal diseases.

Nanoemulsions (NEs) are nanometer droplets made by the heterogeneous dispersions of two immiscible liquids (oil-in-water or water-in-oil) to provide a transparent ocular drug delivery system [129]. NEs are unique as they can solubilize both hydrophobic and hydrophilic drugs to improve the stability, half-life and therapeutic efficacy of the drug delivery [130,131]. Moreover, they provide a large interfacial area compared to the small droplet size [132]. For example, lecithins have been used as the major emulsifiers in the preparation of ocular nanoemulsions [133].

The discovery of nanoemulsions has led to the marketing of several drugs in ophthalmology. In 2002, the first FDA approval was awarded to ophthalmic nanoemulsion of Restasis (Allergan Inc., Irvine, CA, USA) for chronic dry eye conditions. In 2008, the FDA approved another similar nanoemulsion formulated drug called Durezol (Alcon Laboratories, Fort Worth, TX, USA) for the treatment of ocular inflammation. Similarly, two other products, a drug-free nanoemulsion called Lipimix (Tubilux Pharma, Italy) and Soothe XP Emollient (Bausch and Lomb, Rochester, NY, USA), have been used for the restoration of the lipid layer of the lachrymal fluid.

**Table 1:** List of antimicrobials-loaded liposomal formulations reported in the literature.

Antimicrobials	Liposome properties		Experiments	Results	Ref.
	Lipid Composition	Particle size (nm)			
Itraconazole	PC:Chol:SA (7:2:1)	276.5	Rabbit model of microbial keratitis (Strains: Aspergilus flavus URM 6029)	Liposomal formulations decreased fungal burden compared to free drug	[134]
Fluconazole	N/A	N/A	Rabbit model of microbial keratitis (Strains: *C. albicans*)	Complete healing occurred in 86% animals given liposomal formulations compared to 50% in the free drug group. Decreased instillation frequency, duration of recovery and healing compared to free drug	[135]
Tobramycin	Multivesicular liposomes	10^3^–10^5^	Rabbit model of microbial keratitis (Strains: *P. aeruginosa)*	Liposomal formulations combined with fibrin sealants require 5-fold less tobramycin compared to eye drops	[136]
Tobramycin	Hexadecylphosphate (1:2) (Solid-lipid NPs)	80	*In vivo* pharmaco kinetics in rabbits	SLN increases the bioavailability of tobramycin compared to commercial eye drops	[137]
Gentamycin	Phosphatidic acid, PC, a-tocopherol (1:19:0.22)	100–1000	*In vivo* pharmacokinetics by subconjunctival injections in pigmented rabbits	Gentamycin availability increased in the cornea	[138]
Ciprofloxacin	PC:Cho:DODAB	530 ± 25	*In vivo* pharmacokinetics in rabbits	Higher AUC and 3-fold enhanced bioavailability for the liposomal formulations compared to eye drop instillations	[139]
	DPPC:Cho:DODAB	619 ± 71			
	DMPC	580 ± 197			
Ciprofloxacin	PC:Chol (5:3)	1630	*Ex vivo* corneal permeability in rabbits	3-fold increase in transcorneal permeation was observed compared to free drug. Addition of carbopol increased the transcorneal efficiency by about 5 times compared to eye drop	[140]
	PC:Chol :SA (5:3:1)	1850			
	PC:Chol :SA (5:3:1) coated with carbopol gel	–			
Ciprofloxacin	Lecithin:Cho (7:2)	338	*In vitro* antimicrobial assays	Two different liposomal formulations (MLV and REV) of cioprofloxacin were coated onto contact lenses and the MLVs coated lenses showed better zone of inhibition compared to the REVs-coated lenses	[141]
Norfloxacin	DMPC	1090	*Ex vivo* corneal permeability in porcine	Corneal retention of norfloxacin increased for DSPC liposomes	[142]
	DPPC	1410			
	DSPC	2230			

PC–phosphatidylcholine; DPPC–Dipalmitoylphosphatidylcholine; DMPC–Dimyristoyl-sn-glycero-3-phosphocholine; DSPC–distearoyl-L-alpha-phosphatidylcholine; DODAB–Dioctadecyldimethylammonium bromide; SA–Stearylamine; Cho–cholesterol; SLN–solid lipid nanoparticles; MLV–multilamellar vesicles; REV–reverse phase evaporation vesicles.

## 4. Future Directions

Recent progress in nanotechnology to design and engineer nanoparticles is poised to revolutionize the way we diagnose, monitor and treat corneal diseases. It may eliminate the need for repeated applications to achieve sustained drug effect. Though very promising, there remain many challenges to be overcome. One of the most important challenges for the development of ideal therapeutic NPs is the rapid clearance during systemic delivery. Therefore, the factors that affect physicochemical properties, long-term stability, low toxicity and targeted delivery should be considered for future generation of therapeutic nanoparticles. The idea of “Theragonostics” [143] where nanoparticles deliver therapy and provide disease monitoring is very attractive for corneal disease management and vision restoration [144]. The future of corneal nanomedicine greatly depends on the innovative design and smart packaging of nanoparticles better suited for sustained drug-delivery in the eye without compromising the normal functions of the eye tissues. 

## 5. Conclusions

Despite the recent advancements in the field of nanomedicine, there exist no ideal nanoparticulate systems or formulations for the treatment of corneal diseases. Intense research is required to overcome challenges such as particle size, large-scale sterile preparations, multi-ligation, safety and stability of the nanoconstructs. Ongoing research in novel nanotechnologies is expected to overcome these and other hurdles, and it will pave the way to the development of personalized nanomedicine modalities for curing corneal diseases. The interdisciplinary collaborations among scientists from physical, life and medical sciences are key to accelerating this process. 
